# Cdk5 Deletion Enhances the Anti-inflammatory Potential of GC-Mediated GR Activation During Inflammation

**DOI:** 10.3389/fimmu.2019.01554

**Published:** 2019-07-10

**Authors:** Pauline Pfänder, Miray Fidan, Ute Burret, Lena Lipinski, Sabine Vettorazzi

**Affiliations:** Institute of Comparative Molecular Endocrinology (CME), University of Ulm, Ulm, Germany

**Keywords:** inflammation, glucocorticoid receptor, macrophage, Cdk5, Mkp1, p38Mapk, iNos

## Abstract

The suppression of activated pro-inflammatory macrophages during immune response has a major impact on the outcome of many inflammatory diseases including sepsis and rheumatoid arthritis. The pro- and anti-inflammatory functions of macrophages have been widely studied, whereas their regulation under immunosuppressive treatments such as glucocorticoid (GC) therapy is less well-understood. GC-mediated glucocorticoid receptor (GR) activation is crucial to mediate anti-inflammatory effects. In addition, the anti-cancer drug roscovitine, that is currently being tested in clinical trials, was recently described to regulate inflammatory processes by inhibiting different Cdks such as cyclin-dependent kinase 5 (*Cdk5*). *Cdk5* was identified as a modulator of inflammatory processes in different immune cells and furthermore described to influence GR gene expression in the brain. Whether roscovitine can enhance the immunosuppressive effects of GCs and if the inhibition of *Cdk5* affects GR gene regulatory function in innate immune cells, such as macrophages, has not yet been investigated. Here, we report that roscovitine enhances the immunosuppressive Dexamethasone (Dex) effect on the inducible nitric oxide synthase (iNos) expression, which is essential for immune regulation. *Cdk5* deletion in macrophages prevented iNos protein and nitric oxide (NO) generation after a combinatory treatment with inflammatory stimuli and Dex. *Cdk5* deletion in macrophages attenuated the GR phosphorylation on serine 211 after Dex treatment alone and in combination with inflammatory stimuli, but interestingly increased the GR-dependent anti-inflammatory target gene dual-specificity phosphatase 1 (Dusp1, Mkp1). Mkp1 phosphatase activity decreases the activation of its direct target p38Mapk, reduced iNos expression and NO production upon inflammatory stimuli and Dex treatment in the absence of *Cdk5*. Taken together, we identified *Cdk5* as a potential novel regulator of NO generation in inflammatory macrophages under GC treatment. Our data suggest that GC treatment in combination with specific Cdk5 inhibtior(s) provides a stronger suppression of inflammation and could thus replace high-dose GC therapy which has severe side effects in the treatment of inflammatory diseases.

## Introduction

Acute and chronic inflammatory diseases characterized by excessive cytokine and nitric oxide (NO) generation are frequently treated with glucocorticoids (GCs) despite their negative effects including osteoporosis, insulin resistance, muscle atrophy, and depression (1). Adrenalectomized mice failed to survive endotoxic shock without the supplementation of exogenous GCs (2, 3). GCs act through the glucocorticoid receptor (GR or NR3C1), a ligand activated transcription factor, that translocates upon dissociation of accessory proteins (heat shock proteins and immunophilins) into the nucleus and acts either as a monomer or a homodimer to transrepress or transactivate target genes (4, 5). Both mechanisms are crucial to reduce the inflammatory processes either by the GR monomer interacting with pro-inflammatory transcription factors such as nuclear factor kappa B (NF-κB), activator protein 1 (AP-1) or interferon regulatory factor 3 (IRF3) or by the GR homodimer to induce genes that mediate anti-inflammatory effects like GC-induced leucine zipper (*Gilz*) or dual-specificity phosphatase 1 (*Dusp1*, also known as Map kinase phosphatase1, *Mkp1*). GC-induced Mkp1 inhibits inflammatory signaling pathways by dephosphorylation of p38Mapk or c-Jun N-terminal kinase (Jnk) (6, 7). Furthermore, *Gilz*, which is another GR target gene, inhibits NF-κB function in macrophages and T-cells (8–10). Macrophages are one of the first innate immune response cells, and are therefore important targets for the immunosuppressive effect of GC-mediated GR activation. Mice lacking the GR in macrophages show decreased survival during lipopolysaccharide (LPS)-induced endotoxic shock (11). Furthermore, the activation of the GR in macrophages is essential to limit pro-inflammatory cytokine production via Mkp1-mediated p38Mapk inhibition (7). In addition, GCs have been shown to mediate their anti-inflammatory effects during contact hypersensitivity and inflammatory lung injury through the GR in macrophages (12, 13).

Recently, inhibition of cyclin-dependent kinases (Cdks) was found to regulate inflammatory processes by inducing apoptosis in polymorphonuclear leukocytes (PMNs) (14). Roscovitine (Seliciclib, CYC202) is a potent Cdk inhibitor for Cdc2, Cdk2, Cdk5, Cdk7, and Cdk9 (15, 16). Inhibition with this small molecule inhibitor is known to promote apoptosis in cancer cell lines (17). *In vivo*, roscovitine has a potent anti-inflammatory effects during lung inflammation caused by either lipoteichoic acid (LTA) or *Streptococcus pneumoniae* and reduce PMN numbers in bronchoalveolar lavage fluid (18). In inflammatory models, such as bleomycin-induced lung injury and serum transfer-induced arthritis, roscovitine enhances the resolution of the inflammation by either decreasing the anti-apoptotic protein Mcl-1, promoting neutrophil apoptosis or reducing macrophages/monocyte numbers (19, 20). Moreover, the anti-inflammatory role of roscovitine was substantiated by studies with high doses in the RAW264.7 macrophage cell line, which identified a suppression of LPS-induced inducible nitric oxide synthase (*iNos*) expression and nitrite (N${\text{O}}_{2}^{-}$) production, as well as Interleukin-1β (*Il-1*β), Interleukin-6 (*Il-6*), and Tumor necrosis factor-α (*Tnf-*α) mRNA levels (21, 22). Beyond roscovitine, GC-mediated GR activation in LPS-stimulated macrophages is known to reduce *Il-1*β, *Il-6, Tnf*-α, *iNos* mRNA levels, and nitrite (7, 11). Whether the inhibition of Cdks by roscovitine synergistically enhances the anti-inflammatory effects of GC-mediated GR activation in macrophages has not been investigated to date and could be a new therapeutic approach in the treatment of inflammatory diseases.

Roscovitine is likely to act predominantly immunosuppressive through inhibition of cyclin-dependent kinase 5 (*Cdk5)* as it has the highest affinity for this Cdk (23). *Cdk5* is a unique member of the *Cdk* family that was first described to play a pivotal role in the central nervous system (CNS), where it is involved in the regulation of brain development (24, 25), actin dynamics (26), microtubule stability (27, 28), axon guidance (29), and membrane transport (30–32). Beside its expression and function in the brain, *Cdk5* is expressed in immune cells such as neutrophils and T-cells and was shown to be involved in the regulation of neutrophil degranulation and T-cell activation (33, 34). Furthermore, the role of *Cdk5* and its activator p35 (*Cdk5r1*) was investigated in toll-like receptor (TLR)-stimulated primary macrophages. Either *Cdk5* knockdown or p35 knockout led to an increase of Interleukin-10 (Il-10) production by macrophages and resulted in immunosuppression (35). Furthermore, in a model of dextran sulfate sodium (DSS)-induced colitis and sepsis, p35-deficient mice were associated with an enhanced generation of Il-10 (35). The authors report that pro-inflammatory macrophages potentiate inflammation through *p35* and *Cdk5* activation, suggesting that Cdk5 inhibition in macrophages could lower their inflammatory potential (35).

Previous studies have shown that roscovitine also inhibits other Cdks, therefore we were interested in understanding whether the anti-inflammatory effect is mediated by specific inhibition of Cdk5. A link between Cdk5 and GR was reported by two studies in rat neuronal cells as well as in the prefrontal cortex and hippocampus of stress exposed mice showing that Cdk5 phosphorylates the GR at different serine residues and therefore modulates the GR transcriptional activity in the brain (36, 37). However, the role of *Cdk5* in combination with GCs mediated-GR activation in macrophages under inflammatory conditions has not been investigated to date.

Here, we report that roscovitine, a pan-Cdk inhibitor, as well as specific *Cdk5* deletion in macrophages enhance the anti-inflammatory effect of GCs. The treatment with Dexamethasone (Dex), a synthetic GC, in combination with roscovitine synergistically suppresses iNos mRNA and protein expression after LPS induction in bone marrow derived macrophages (BMDMs). *Cdk5* deletion confirmed a synergistic Dex-mediated suppression of iNos mRNA and protein as well as NO generation in inflammatory macrophages. However, roscovitine showed also in the absence of Cdk5 a synergistic effect with Dex to a certain degree mediated by the inhibition of other Cdks than Cdk5. This indicates that roscovitine enhances the anti-inflammatory Dex effect on iNos by inhibiting Cdk5 and other Cdks. However, Dex-mediated suppression of pro-inflammatory cytokines such as *Il-1*β and *Il-6* was not enhanced by roscovitine treatment or *Cdk5* deletion. In addition, the effect on iNos and NO production was associated with decreased phosphorylation of GR (Ser211), but interestingly induced expression of GR target gene Mkp1 and reduced p38Mapk activation in *Cdk5* deficient macrophages. The reduction of p38Mapk activity further enhanced the Dex effect on iNos repression. These results show that inhibition of Cdk5, in combination with Dex treatment improves the suppression of iNos and NO in macrophages. Inhibiting Cdks by roscovitine and/or specific impairing Cdk5 activity could serve as a new treatment strategy in high-dose GC therapy of inflammatory diseases.

## Materials and Methods

### Mice

*Cdk5*^tm1Bibb^ (C57BL/6) mice (hereafter named as *Cdk5*^flox^) were kindly provided by Prof. Dr Johanna Pachmayr (Paracelsus Medical Private University, Austria) (38). *Cdk5*^flox^ mice were crossed with transgenic Lyz2^tm1(cre)lfo/J^ (C57BL/6) mice (hereafter named as LysMCre) to generate *Cdk5*^LysMCre^ mice. Male and female *Cdk5*^LysMCre^ mice and littermate controls (*Cdk5*^flox^) at the age of 8–13 weeks were used for experiments. The mice were genotyped by PCR using genomic DNA isolated from the tails. All animals were housed under specific pathogen-free conditions at the Centre of Biomedical Research (ZBMF) at Ulm University. This study was carried out in accordance with the recommendations of Tierschutzgesetz and Tierschutz-Versuchstierordnung (Mitteilung nach § 4), Regierungspräsidium Tübingen, Baden-Württemberg. The protocol was approved by the Regierungspräsidium Tübingen, Baden-Württemberg.

### Cell Culture

The primary BMDMs were isolated from humerus, femur and tibia of 8–13 weeks old mice as described previously (11). Briefly, cells were cultured until day 7 in DMEM (D5671, sigma) supplemented with 10% fetal bovine serum (FBS, F7524, sigma), 20% L929-cell conditioned medium, 1% Penicillin/Streptomycin (P0781, sigma), 1% L-Glutamine (G7513, sigma), 1% Sodium Pyruvate (S8636, sigma) at 37°C, and 5% CO_2_. For roscovitine experiments, BMDMs from wildtype mice (C57BL/6) or from *Cdk5*^flox^ and *Cdk5*^LysMCre^ were pre-treated for 30 min with DMSO (as vehicle) or 10 μM roscovitine (Seliciclib, CYC202) (Selleckchem). For p38Mapk inhibition, BMDMs from wildtype mice (C57BL/6) were pre-treated for 1 h with DMSO or 5 μM SB203580 (sigma). BMDMs were isolated from littermate wildtype (*Cdk5*^flox^) and *Cdk5*^LysMCre^ mice. All BMDMs were treated with PBS as control, LPS (100 ng/ml, L6529, sigma), Dex (10^−6^ M, D2915, sigma), or LPS + Dex (100 ng/ml LPS and 10^−6^ M Dex) for the indicated durations. For alternative macrophage (M2-like) polarization as well as for TAM and phagocytic receptor expression analysis, cells were treated 24 h as indicated with PBS as control, Il-4 (20 ng/ml, Immunotools), Il-13 (20 ng/ml, Immunotools), Il-4 + Il-13 (20 ng/ml Il-4 and 20 ng/ml Il-13), Il-10 (20 ng/ml, Immunotools), Il-10 + Dex (20 ng/ml Il-10 and 10^−7^ M Dex), Dex (10^−7^ M), or LPS + Dex (100 ng/ml LPS and 10^−7^ M Dex).

### NO Measurement

Bone marrow derived macrophages were isolated from *Cdk5*^flox^ and *Cdk5*^LysMCre^ mice and grown until day 6. Afterwards, cells were seeded in a 96-well plate (150'000 cells/well) with DMEM media without phenolred (D1145, sigma) supplemented with 10% fetal bovine serum (FBS, F7524, sigma), 20 ng/ml M-CSF (R&D system), 1% Penicillin/Streptomycin (P0781, sigma), 1% L-Glutamine (G7513, sigma), 1% Sodium Pyruvate (S8636, sigma), and incubated at 37°C and 5% CO_2_. At day 7, BMDMs were treated for the indicated time points. Supernatant was collected after 48 h, centrifuged (13'000 rpm, 5 min) and nitrite was measured as a stable metabolite of NO with Griess reagent (Molecular Probes; G7921) according to the manufacturer's protocol.

### ELISA

For the determination of Il-6 secretion, the medium of BMDMs from littermate wildtype (*Cdk5*^flox^) and *Cdk5*^LysMCre^ mice after 4 h treatment with PBS as control, LPS (100 ng/ml), Dex (10^−6^ M) or LPS + Dex (100 ng/ml LPS and 10^−6^ M Dex) was collected, sterile filtered (0.2 μm) and stored at −80°C until measurement was performed. The Il-6 ELISA was performed with the Mouse Il-6 ELISA set (BD OptEIA^™^) according to the manufacturer's protocol. The absorption was measured using the Dynex Opsys MR 96-Well Microplate Reader at 405 nm with a correction wavelength of 650 nm.

### RNA Isolation and Quantitative RT-PCR

Primary macrophages were washed with 1x PBS and then scraped in RLT (Qiagen) + 10 μl β-mercaptoethanol/ml buffer. RNA was isolated using the RNeasy^®^ Mini Kit (Qiagen) according to the manufacturer's protocol. Next, 1,000 ng RNA was reversed transcribed to cDNA by using Superscript II^®^ (Superscript^®^ Reverse Transcriptase, Invitrogen). Quantitative RT-PCR (qRT-PCR) was performed with the ViiA^™^ 7 Realtime PCR System (Life technologies) using Platinum SYBR Green (Invitrogen). For analysis the QuantStudio Realtime-PCR software and the ΔΔCT method was used. β*-Actin* and *Ribosomal protein L* (*Rpl)* served as housekeeping genes. The specific primers were obtained from Sigma with the sequences as listed below:

**Table:** Primer Sequences

**Gene**	**Forward Primer (5^**′**^ → 3^**′**^)**	**Reverse Primer (3^**′**^ → 5^**′**^)**
*β-Actin*	GCACCAGGGTGTGATGGTG	CCAGATCTTCTCCATGTCGTCC
*Anxa1*	AAGGTGTGGATGAAGCAACC	AGGGCTTTCCATTCTCCTGT
*Axl*	AGCCTTCCTGTGCCCCTA	GAGGTGGGGGTTCACTCA
*Cd36*	TGGCAAAGAACAGCAGCAAA	CACAGTGTGGTCCTCGGG
*Cd163*	GGCTAGACGAAGTCATCTGCAC	CTTCGTTGGTCAGCCTCAGAGA
*Cd206*	CCACAGCATTGAGGAGTTTG	ACAGCTCATCATTTGGCTCA
*Cdk5*	TGGACCCTGAGATTGTGAAGT	GACAGAATCCCAGGCCTTTC
*Gilz*	ACCAGACCATGCTCTCCATT	GGCCTGCTCAATCTTGTTGT
*Il-1β*	GGCTGTGGAGAAGCTGTGGCA	GGGTCCGACAGCACGAGGCT
*Il-6*	AAACCGCTATGAAGTTCCTCTCTGC	AGCCTCCGACTTGTGAAGTGGT
*Il-10*	CAGAGCCACATGCTCCTAGA	TGTCCAGCTGGTCCTTTGTT
*iNos*	CTGCTTTGTGCGAAGTGTCAGT	GGCACCCAAACACCAAGCTC
*Mertk*	GCTGGCATTTCATGGTGGAA	CATTGTCTGAGCGCTGCAC
*Mkp1*	GTGCCTGACAGTGCAGAATC	CACTGCCAGGTACAGGAAG
*Rpl*	CCTGCTGCTCTCAAGGTT	TGGCTGTCACTGCCTGGTACTT
*Tyro3*	TGGAGCCATCCTAGAGTTCC	GAGGGGCCTGACTTCCTG
*Ym1*	CTGGGTCTCGAGGAAGCC	AGTGAGTAGCAGCCTTGGAA

### Western Blot Analysis

Bone marrow derived macrophages were washed with 1x PBS and lysed directly on the dishes with ice-cold 1x Lysis Buffer (Cell Signaling) or 1x RIPA buffer. PhosphoStop (Roche) and protease inhibitor cocktail (Roche) were added to both buffers. The lysates were centrifuged at 14'000 rpm at 4°C for 10 min. The protein concentration was determined using the Pierce^®^ BCA Protein Assay Kit (Thermo Scientific) according to the manufacturer's instructions. For Western blot analysis, protein samples were adjusted to 15–35 μg protein with Lysis or RIPA buffer and boiled in 5x Laemmli buffer (with 10 μl/ml β-mercaptoethanol) at 95°C for 5 min. Equal protein amounts were separated on 7.5–10 % SDS—PAGE gels and subsequently electrotransferred onto nitrocellulose membranes (Biorad) using the Tank Blot System (Biorad). The membranes were blocked with 5% skim milk powder (Fluka Analytical) or BSA (Sigma) in Tris-buffered saline with Tween20 (TBS-T) for 1 h at RT and probed over night at 4°C with primary antibodies against β-Actin (Sigma Aldrich), Cdk5 (Cell Signaling #2506), GR (Cell Signaling #12041), phospho-GR Ser211 (Cell Signaling #4161), iNos (Santa Cruz Biotechnology sc-650 or Cell Signaling #13120), p38Mapk (Cell Signaling #9218), phospho-p38Mapk Thr180/Tyr182 (Cell Signaling #4511), Mkp1 (Santa Cruz Biotechnology sc-871684). After washing with TBS-T for 30 min, membranes were incubated with horseradish peroxidase-coupled goat anti-mouse (Dako) or goat anti-rabbit (Life technologies) antibodies for 1 h at RT. For visualization the LuminataTM Forte Western HRP Substrate (Milipore) and the ChemiDocTM MP Imaging System (Biorad) was used. If membranes were stripped, blots were incubated with stripping buffer (with 0.5 μl β-mercaptoethanol/ml) at 60°C for 30 min. Phospho-proteins were always first detected and total protein after stripping. Quantification was performed with Photoshop software. Cdk5, iNos, and Mkp1 were normalized to β-Actin as loading controls. Phospho-p38Mapk was normalized to p38Mapk as loading control. pGR was normalized to β-Actin on the same gel and total GR was normalized to β-Actin on the same gel and afterwards p-GR/GR ratio was calculated.

### Multiplex-Assay

Phospho-Erk1/2 (Thr202/Tyr204) protein was detected with the Bio-Plex Pro^™^ cell signaling MAPK-Panel (#LQ00000S6KL81S, Biorad). The Bio-Plex Assay was conducted according to the manufacturer's protocol. The median fluorescence intensity (MFI) was detected with the Bio-Plex 200 machine (Biorad) and analyzed with the Bio-Plex Manager^™^ 6.1 software (Biorad).

### Statistical Analysis

Statistical analysis was carried out with GraphPad Prism 7 software. All data are shown as mean ± SEM. Outlying sample exclusion criteria were done with GraphPad Prism Outlier Calculator. All data were tested using the Wilcoxon-Mann-Whitney test (two-tailed). In comparison, mean values which show significance are indicated as follows: ^*^p < 0.05; ^**^p < 0.01; ^***^p < 0.001; ^****^p < 0.001; ns: not significant.

## Results

### Roscovitine Enhances the Dex-Mediated Suppression of iNos in Inflammatory Macrophages

**Figure 1:**
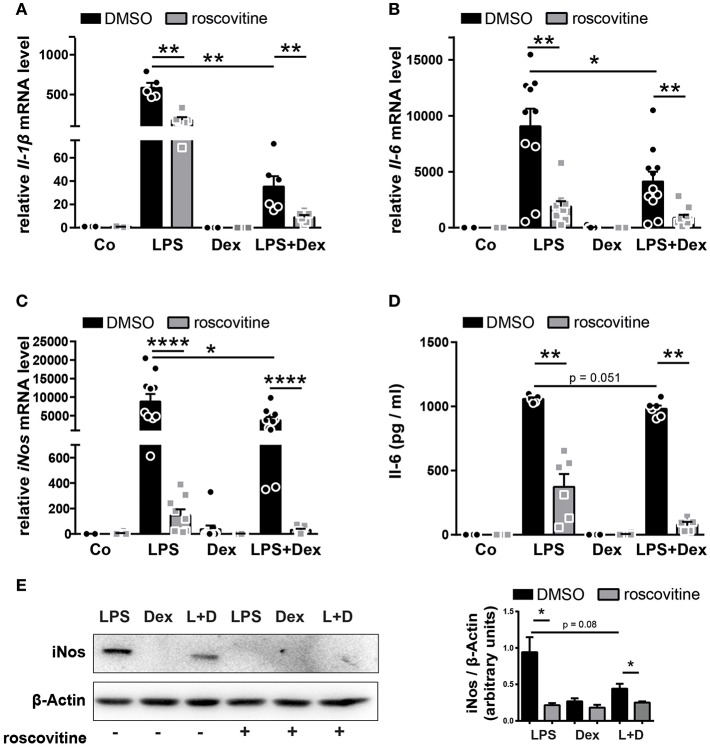
The combination of roscovitine and Dex shows additive and synergistic anti-inflammatory effects in inflammatory macrophages. **(A–E)** BMDMs derived from wildtype mice were stimulated 4 h with PBS (Co), LPS (100 ng/ml), Dex (10^−6^ M), or a combination of LPS + Dex (L + D) with 30 min pre-treatment of either DMSO or 10 μM roscovitine. **(A)** Relative *Il-1*β mRNA expression, **(B)** relative *Il-6* mRNA expression, and **(C)** relative *iNos* mRNA expression were analyzed by qRT-PCR after 4 h. **(D)** Il-6 protein concentration was determined in the BMDM supernatant by ELISA after 4 h. **(E)** BMDMs derived from wildtype mice were stimulated 4 h with LPS (100 ng/ml), Dex (10^−6^ M), or a combination of LPS + Dex (L + D) with 30 min pre-treatment of either DMSO or 10 μM roscovitine and iNos protein (130 kDa) was detected by western blot after 4 h and quantified. β-Actin (43 kDa) served as loading control. Data shown in **(A)**: *n* = 5–6; **(B)**: *n* = 11–12; **(C)**: *n* = 9–11; **(D)**: *n* = 5–6; and **(E)**: *n* = 3. Results are depicted as mean ± SEM. Statistical analysis was performed by Wilcoxon-Mann-Whitney test (two-tailed) ^*^p < 0.05; ^**^p < 0.01; ^****^p < 0.0001.

To examine whether roscovitine enhances the immunosuppressive effects of GCs we first determined the expression levels of pro-inflammatory cytokines in BMDMs isolated from wildtype mice. Since iNos expression and ultimately NO production in macrophages are essential for immune regulation during inflammation, we further examined iNos expression. As expected from previous studies (7, 11), 4 h LPS stimulation upregulated the expression of *Il-1*β, *Il-6*, and *iNos* mRNA and iNos protein, whereas Dex treatment during LPS stimulation significantly reduced their expression, and Il-6 protein showed a trend in reduction (Figures 1A–E). Moreover, as previously shown (19), the LPS-mediated induction of inflammatory mediators *Il-1*β, *Il-6*, and iNos was significantly decreased upon solely roscovitine treatment both on the mRNA and protein levels (Figures 1A–E). Interestingly, the combinatorial treatment of Dex with roscovitine during LPS stimulation further reduced the *Il-1*β, *Il-6*, and *iNos* mRNA and Il-6 and iNos protein levels (Figures 1A–E). To investigate whether the strong reduction in inflammatory mediator expression is mediated by either additive or synergistic anti-inflammatory effects of roscovitine and Dex, we calculated the degree of the suppressive Dex effect with and without roscovitine by setting LPS treatment to 100%. We found that the Dex-mediated suppression of the pro-inflammatory cytokines *Il-1*β and *Il-6* was not enhanced by roscovitine treatment in inflammatory macrophages, suggesting that the immunosuppressive roscovitine effect is mediated by independent pathways (Figures 1A,B and Supplementary Figures 1A,B). However, roscovitine increased the anti-inflammatory potential of Dex on LPS-induced *iNos* expression (Figures 1C,E and Supplementary Figure 1C). These results suggest that roscovitine has a strong immunosuppressive effect that is enhanced when given in combination with Dex, further reducing inflammatory mediator expression in macrophages. Interestingly, in the case of iNos expression, roscovitine enhances the anti-inflammatory potential of Dex in a synergistic manner.

### Specific Cdk5 Deletion Enhances the Suppressive Dex-Effect on iNos in Inflammatory Macrophages

Roscovitine shows highest affinity for *Cdk5* (15, 16) therefore we assumed that the immunosuppressive effects of roscovitine are mainly due to the inhibition of *Cdk5*. *Cdk5* has primarily been implicated in brain development and is particularly important in neuronal maturation and migration (24–31). It also has been reported to be expressed in immune cells like macrophages with a functional relevance *in vitro* and *in vivo* (33–35). Since we observed a difference on the anti-inflammatory Dex effect upon combinatorial treatment with roscovitine in LPS-stimulated BMDMs, we further investigated under the same conditions the impact of *Cdk5* deficiency on inflammatory processes in macrophages. Thus, we used the Cre/loxP-system and crossed *Cdk5*^flox^ mice with myeloid specific lysozyme MCre mice (Lyz2^tm1(cre)Ifo^, hereafter named as LysMCre) (39). The BMDMs isolated from the mutant mice (*Cdk5*^LysMCre^) showed a significant decrease in *Cdk5* at the mRNA and protein levels (Supplementary Figures 2A,B). Thus, *Cdk5*^LysMCre^ mice serve as a suitable model to study specific *Cdk5* effects in macrophages.

**Figure 2:**
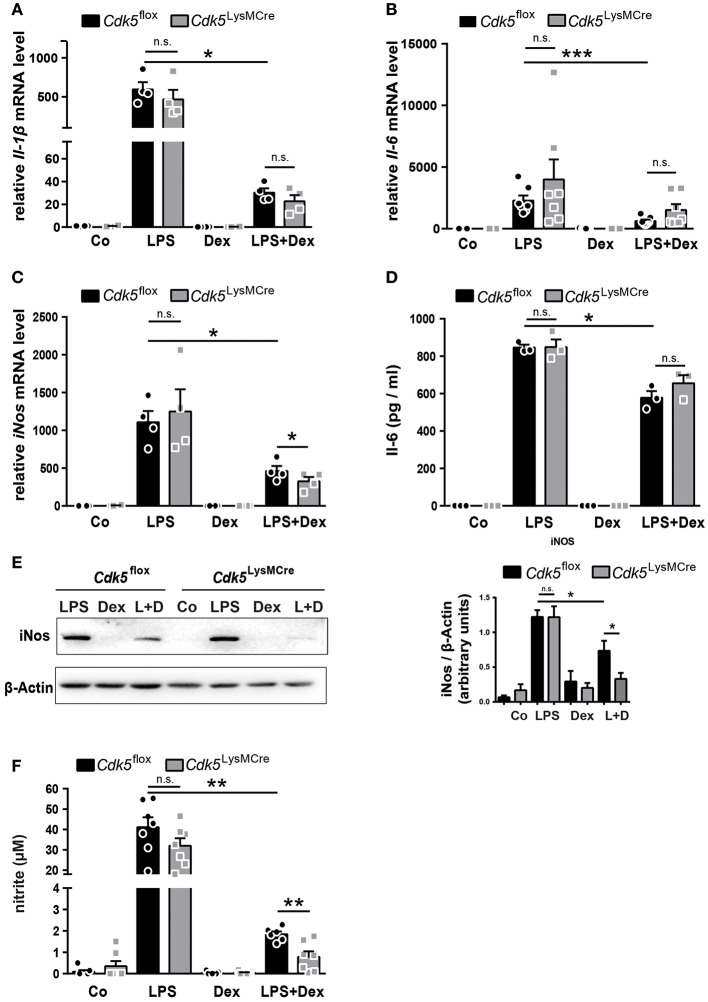
*Cdk5* deletion has no impact on inflammatory mediators but potentiates the Dex effect on iNos and NO production in macrophages. **(A–E)** BMDMs from *Cdk5*^flox^ and *Cdk5*^LysMCre^ mice were stimulated with PBS (Co), LPS (100 ng/ml), Dex (10^−6^ M), or a combination of LPS + Dex (L + D) for 4 h. **(A)** Relative *Il-1*β mRNA expression, **(B)** relative *Il-6* mRNA expression, and **(C)** relative *iNos* mRNA expression were analyzed by qRT-PCR after 4 h. **(D)** Il-6 protein concentration was determined by ELISA in the supernatant of *Cdk5*^flox^ and *Cdk5*^LysMCre^ BMDMs after 4 h. **(E)** BMDMs from *Cdk5*^flox^ and *Cdk5*^LysMCre^ mice were stimulated with LPS (100 ng/ml), Dex (10^−6^ M) or a combination of LPS + Dex (L+D) for 4 h and iNos protein (130 kDa) was detected by western blot after 4 h and quantified. β-Actin (43 kDa) served as loading control. **(F)** BMDMs were treated as described in A and nitrite (a stable NO metabolite) was measured in the supernatant of *Cdk5*^flox^ and *Cdk5*^LysMCre^ BMDMs after 48 h. Data shown in **(A)**: *n* = 4; **(B)**: *n* = 7; **(C)**: *n* = 4; **(D)**: *n* = 3; **(E)**: *n* = 5–8; **(F)**: *n* = 6–7. Results are depicted as mean ± SEM. Statistical analysis was performed by Wilcoxon-Mann-Whitney test (two-tailed) ^*^p < 0.05; ^**^p < 0.01; ^***^p < 0.001; n.s. not significant.

As expected, the mRNA expression of *Il-1*β, *Il-6* and *iNos* as well as Il-6 and iNos protein expression were increased after 4 h of LPS stimulation and significantly reduced by Dex after LPS induction in *Cdk5*^flox^ BMDMs (Figures 2A–E). However, *Cdk5* deletion did not reduce *Il-1*β, *Il-6*, and *iNos* mRNA as well as Il-6 and iNos protein expression after single LPS stimulation (Figures 2A–E). This is in contrast to our observation upon roscovitine treatment (Figures 1A–E), suggesting that the strong immunosuppressive effect of roscovitine on inflammatory mediators is not solely mediated by the inhibition of Cdk5 but by the inhibition of several Cdks. To determine whether the observed synergistic effect of roscovitine and Dex on *iNos* suppression, but not on *Il-1*β and *Il-6*, is mediated by inhibition of Cdk5 we investigated the *Il-1*β, *Il-6*, and *iNos* expression in BMDMs isolated from wildtype (*Cdk5*^flox^) and *Cdk5*^LysMCre^ mice. The *Cdk5* deletion had no effect on the anti-inflammatory potential of Dex on *Il-1*β an *Il-6* expression (Figures 2A,B and Supplementary Figures 1D,E). However, *Cdk5* deletion enhanced significantly the Dex-mediated downregulation of *iNos* expression after 4 h of LPS induction in comparison to wildtype BMDMs (Figure 2C and Supplementary Figure 1F). Furthermore, we found that *Cdk5* deletion significantly enhanced the suppressive Dex effect also on the iNos protein level after 4 h of LPS + Dex stimulation (Figure 2E), followed by a stronger reduction of NO production in the supernatant of *Cdk5*^LysMCre^ BMDMs (Figure 2F). This suggests that the enhanced Dex effect in the presence of roscovitine may be mediated by the inhibition of Cdk5. To further confirm the enhanced anti-inflammatory Dex effect by roscovitine and *Cdk5* deletion, we investigated the effect of roscovitine in *Cdk5*^LysMCre^ macrophages. Roscovitine reduced *Il-1*β, *Il-6*, and *iNos* expression in LPS treated macrophages in the absence of Cdk5 (Supplementary Figures 3A–C), showing that the anti-inflammatory potential of roscovitine is mediated mainly by the inhibition of other kinases than Cdk5. Moreover, we observed that roscovitine treatment of LPS + Dex treated *Cdk5*^LysMCre^ macrophages reduced the expression of *Il-1*β and *Il-6*, suggesting that these effects are mediated by Cdk5-independent pathways (Supplementary Figures 3A,B,D,E). Interestingly, *iNos* expression was also reduced in LPS + Dex treated *Cdk5*^LysMCre^ macrophages when roscovitine was present (Supplementary Figures 3C,F). This suggests that roscovitine enhances the anti-inflammatory Dex effect on iNos mainly by a Cdk5-independent mechanism.

However, deletion of Cdk5 is sufficient to increase the Dex effect on *iNos*, indicating Cdk5 as an important target to increase anti-inflammatory efficacy of GCs.

### Specific *Cdk5* Deletion Has no Effect on Anti-inflammatory Markers in Macrophages

GCs mediate their anti-inflammatory effects not only by suppressing pro-inflammatory mediators in M1-like macrophages, but also by promoting alternative anti-inflammatory M2-like macrophage polarization. Since we observed a synergistic anti-inflammatory effect on the M1 marker iNos upon *Cdk5* deletion and Dex treatment, we next examined if a combination of *Cdk5* deletion and Dex treatment has an effect on M2 macrophage polarization *in vitro*. Therefore, BMDMs from *Cdk5*^flox^ and *Cdk5*^LysMCre^ were stimulated for 24 h with the M2 stimuli Il-4, Il-10, Il-13, Dex, Il-4 + Il-13, and Il-10 + Dex. We found that *Cdk5* deletion alone had no impact on known typical M2-like markers (*Cd163, Cd206, Ym1*, and *Il-10*) (Supplementary Figure 4). When *Cdk5* deleted BMDMs were treated with Il-10, a trend toward a reduction in expression of *Cd163* was observed (Supplementary Figure 4A). Similarly, no significant changes were observed in the expression of *Cd206* (Supplementary Figure 4B), *Ym1* (Supplementary Figure 4C), and *Il-10* (Supplementary Figure 4D). Furthermore, no *Cdk5* specific effects on M2 marker expression were determined in combination with Dex (Supplementary Figures 4A–D). However, the combination of *Cdk5* deletion and Dex treatment led to an enhanced induction of *Mertk* expression (Supplementary Figure 4E). *Mertk* is a member of the TAM receptor family, which includes Tyro3, Axl, and Mer. These receptors are important for macrophage phagocytic function (40). Therefore, we examined whether *Cdk5* deletion regulates TAM and phagocytic receptor expression in inflammatory macrophages treated with Dex. Our findings revealed that the expression of other phagocytic receptors such as *Tyro3* (Supplementary Figure 4F), *Axl* (Supplementary Figure 4G), *Cd36* (Supplementary Figure 4H), and *Anxa1* (Supplementary Figure 4I) were not altered upon *Cdk5* deletion. Therefore, we concluded that Cdk5 alone as well as in combination with Dex does not play a major role in regulating M2-like markers and TAM receptor expression in macrophages *in vitro*. We interpret these results to mean that *Cdk5* deletion potentiates the suppressive Dex effect on the pro-inflammatory marker iNos and NO production in LPS stimulated BMDMs (Figures 2C,E,F).

### *Cdk5* Deletion Is Associated With a Reduced GR Phosphorylation at Ser211, but Interestingly Increased Induction of the GR Target Gene Mkp1

**Figure 3:**
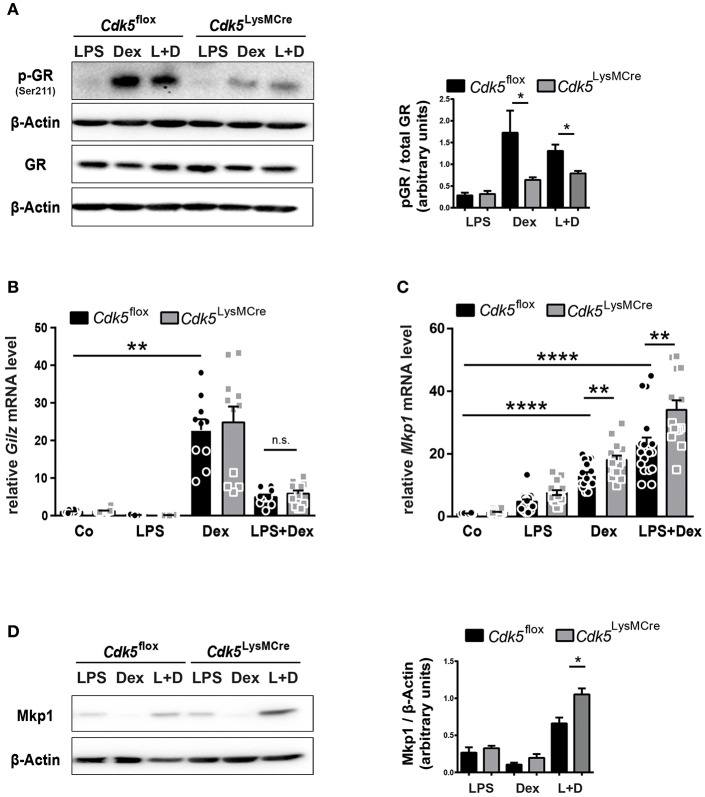
*Cdk5* deletion diminish GR phosphorylation, but increases the GR target gene Mkp1. **(A)** BMDMs derived from *Cdk5*^flox^ and *Cdk5*^LysMCre^ mice were stimulated with LPS (100 ng/ml), Dex (10^−6^ M) or a combination of LPS + Dex (L + D) for 4 h and phosphorylated GR (Ser211) protein (95 kDa) and total GR protein (94 kDa) was detected by western blot on two separate gels and quantified. β-Actin (43 kDa) served as loading control on the individual gels. **(B,C)** BMDMs from *Cdk5*^flox^ and *Cdk5*^LysMCre^ mice were stimulated with PBS (Co), LPS (100 ng/ml), Dex (10^−6^ M), or a combination of LPS + Dex for 4 h and **(B)** relative *Gilz* mRNA expression and **(C)** relative *Mkp1* mRNA expression were measured with qRT-PCR after 4 h. **(D)** BMDMs were treated as described in **(A)** Mkp1 protein (40 kDa) was detected by western blot after 4 h. β-Actin (43 kDa) served as loading control. Data shown in **(A)**: *n* = 3–4; **(B)**: *n* = 10–12; *n* = 14–15; and **(D)**: *n* = 5. Results are depicted as mean ± SEM. Statistical analysis was performed by Wilcoxon-Mann-Whitney test (two-tailed) ^*^p < 0.05; ^**^p < 0.01; ^****^p < 0.0001; n.s. not significant.

The Cdk5-regulated pathways beyond the brain have not been well studied; therefore, we further examined how *Cdk5* deletion enhances the Dex effect on iNos and NO. There are reports showing that Cdk5 can directly interact with the GR by changing the phosphorylation status and thereby influencing GR transcriptional activity (36, 41). It was reported that GR phosphorylation by Cdk5, in particular at serine 211 (Ser211), reduces GR transcriptional activity in the context of neurons (41). We therefore investigated the GR phosphorylation at Ser211 in *Cdk5*^flox^ and *Cdk5*^LysMCre^ BMDMs. Our results demonstrated that Dex and LPS + Dex treatment increased GR phosphorylation at Ser211 in *Cdk5*^flox^ BMDMs after 4 h, whereas deletion of *Cdk5* led to a significant decrease in GR phosphorylation after Dex and LPS + Dex treatment (Figure 3A and Supplementary Figure 5A). In line with this, roscovitine treatment of wildtype macrophages also confirmed a tendency toward a reduced GR Ser211 phosphorylation after Dex and LPS + Dex treatment (Supplementary Figure 5B). Thus, our findings show for the first time that deletion of *Cdk5* diminishes GR phosphorylation at Ser211 in macrophages.

Since GR phosphorylation at Ser211 has been described as an activating phosphorylation site (42), but is known to act also as a suppressive phosphorylation site in neurons (36), thus, we further analyzed GR transcriptional activity upon *Cdk5* deletion. To this end, we tested the expression of the anti-inflammatory target *Gilz* and we did not observe differences irrespective of genotype upon 4 h of Dex stimulation (Figure 3B). *Gilz* is a negative regulator of Raf-Mek1/2-Erk1/2 activation (43) and in line, we observed no changes in Erk1/2 phosphorylation in *Cdk5*^flox^ and *Cdk5*^LysMCre^ BMDMs (Supplementary Figure 5C). Moreover, we detected strikingly reduced GR phosphorylation at Ser211 after 4 h of Dex and LPS + Dex treatment in *Cdk5*^LysMCre^ BMDMs (Figure 3A and Supplementary Figure 5A). However, the anti-inflammatory GR target *Mkp1* was significantly induced in *Cdk5*-deficient BMDMs after 4 h of Dex and LPS + Dex stimulation (Figure 3C). In addition, Mkp1 protein was increased in *Cdk5* deleted BMDMs upon 4 h Dex and LPS + Dex stimulation (Figure 3D and Supplementary Figure 5D). This suggests that also for certain GR target genes in macrophages the phosphorylation at Ser211 is associated with diminished GR transcriptional activity.

### *Cdk5* Deletion Reduces Phospho-p38Mapk and Hence iNos and NO Production During LPS Stimulation and Dex Exposure

**Figure 4:**
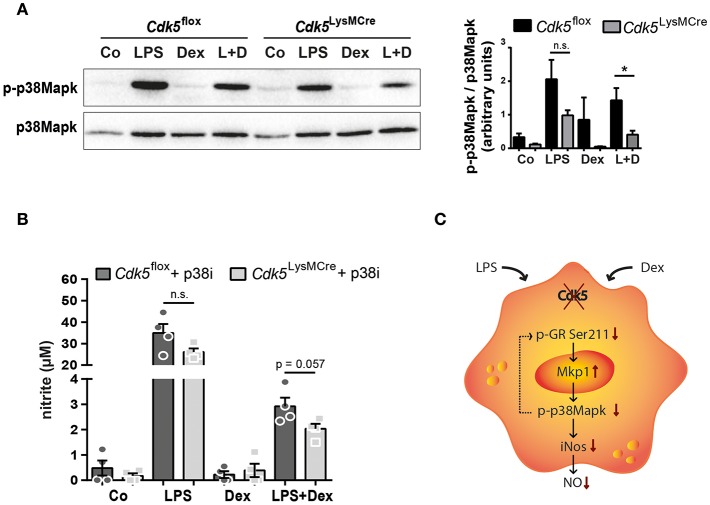
*Cdk5* deletion reduces phospho-p38Mapk leading to decreased NO production. **(A)** BMDMs derived from *Cdk5*^flox^ and *Cdk5*^LysMCre^ mice were stimulated with PBS (Co), LPS (100 ng/ml), Dex (10^−6^ M), or a combination of LPS + Dex (L + D) and phospho-p38Mapk (43 kDa) and p38Mapk (40 kDa) protein were detected by western blot after 4 h and quantified. **(B)** BMDMs were treated as described in **(A)** with 1 h pre-treatment of either DMSO or 5 μM SB203580 (p38Mapk inhibitor = p38i) and nitrite (a stable NO metabolite) was measured in the supernatant after 48 h. **(C)** Scheme showing NO regulation in the absence of *Cdk5* via the GR-Mkp1-p38Mapk axis in macrophages under inflammatory (LPS) conditions and GC (Dex) treatment. Possible reduction of GR (Ser211) phosphorylation by p38Mapk is shown with a dotted arrow. Data shown in **(A)**: *n* = 5–6 and **(B)**: *n* = 4. Results are depicted as mean ± SEM. Statistical analysis was performed by Wilcoxon-Mann-Whitney test (two-tailed) ^*^p < 0.05; n.s. not significant.

Our experimental data revealed that *Cdk5* deletion synergistically reduces LPS-induced iNos expression and NO production in combination with Dex treatment (Figures 2C,E,F). Previous studies have shown that iNos expression is also regulated by Mapk pathways, such as p38Mapk (44), whose activating phosphorylation levels are reduced by an increased Mkp1 expression in response to LPS induction (45). Therefore, we further investigated p38Mapk as a potential link between *Cdk5* regulating iNos and NO production via Mkp1. We demonstrated that 4 h of LPS stimulation increased p38Mapk phosphorylation, whereas Dex treatment attenuated its phosphorylation after LPS stimulation in *Cdk5*^flox^ BMDMs (Figure 4A), as expected (7). Interestingly, *Cdk5* deletion in combination with Dex treatment during inflammatory stimuli attenuated p38Mapk phosphorylation to a greater extent compared to *Cdk5*^flox^ macrophages (Figure 4A). However, similar a previous publication (21), roscovitine treatment of inflammatory wildtype macrophages show unchanged levels of p38Mapk phosphorylation (Supplementary Figure 5E).

To prove whether *Cdk5* deletion enhances the Dex effect on NO production after inflammatory stimuli via the GR-Mkp1-p38Mapk axis, we examined the NO production in the supernatants of *Cdk5*^flox^ and *Cdk5*^LysMCre^ BMDMs after inhibition of p38Mapk. Indeed, we observed a trend toward a potentiated repressive Dex effect on NO production after 48 h of p38Mapk inhibition in LPS + Dex treated *Cdk5*^LysMCre^ BMDMs (Figure 4B). This suggests that a lack of *Cdk5* enhances the suppressive Dex effect through a reduced GR Ser211 phosphorylation and increased *Mkp1* expression that in turn attenuates p38Mapk activation and thus iNos and NO production in inflammatory macrophages (Figure 4C).

## Discussion

In this study we showed that roscovitine exert its function, in contrast to the general view, rather independent of Cdk5. Moreover we presented for the first time that roscovitine as well as *Cdk5* deletion potentiated the anti-inflammatory effect of Dex on iNos and NO production in LPS-stimulated macrophages by mainly two independent mechanisms. We further demonstrated that under inflammatory conditions and Dex treatment GR Ser211 phosphorylation is stronger reduced upon *Cdk5* deletion, whereas the GR transcriptional target gene Mkp1 was induced. An increased expression of Mkp1 phosphatase led to an increased dephosphorylation of p38Mapk, which in turn resulted in a decreased iNos and NO production. Collectively, our findings showed for the first time that macrophage specific *Cdk5* deletion in combination with Dex potentiates the anti-inflammatory effect of GCs on iNos.

### Roscovitine Enhances the Immunosuppressive Dex Effect on iNos Independent of Cdk5

Roscovitine is a small molecule inhibitor that is currently in phase II clinical trials for cancer treatments like Cushing syndrome and non-small cell lung cancer (Clinical trials NCT03774446, NCT00372073). Furthermore, roscovitine treatment reduced lung inflammation and enhanced the resolution of inflammation during arthritis by enhancing apoptosis of neutrophils and favoring phagocytosis by macrophages (18, 20). However, in macrophages, only limited *in vitro* studies have been performed using cell lines (RAW264.7), showing that high concentrations (20 μM) of roscovitine inhibit cell viability after 24 h of treatment (22). In the current study 10 μM roscovitine (for a duration of 4 h) had no phenotypic effect on proliferation and apoptosis in primary macrophages.

We analyzed the inflammatory response of primary macrophages after roscovitine treatment. In line with Du et al. and Jhou et al. roscovitine treatment led to a significant reduction of *Il-1*β, *Il-6*, and *iNos* mRNA expression as well as Il-6 and iNos protein expression after LPS stimulation (21, 22). Cdk5 is a high affinity roscovitine target, and so has been the focus of studies investigating inflammatory diseases such as DSS-induced colitis, LPS-induced endotoxic shock and experimental autoimmune encephalomyelitis (34, 35). However, macrophage specific *Cdk5* deletion did not reduce *Il-1*β*, Il-6, iNos* mRNA, and Il-6 and iNos protein expression after LPS induction, showing that the anti-inflammatory effect of roscovitine is not mediated by Cdk5 inhibition. At high concentrations (10 μM) roscovitine also inhibits other kinases including Cdk1 (IC_50_ = 0.65 μM), Cdk2 (IC_50_ = 0.7 μM), Cdk7 (IC_50_ = 0.46 μM), Cdk9 (IC_50_ = 0.6 μM), and Erk (IC_50_ = 14–34 μM). Therefore, it is likely that the observed effect is mediated by the inhibition of several Cdks (15, 16). A previous study demonstrated that the anti-inflammatory effect of roscovitine on *Il-1*β, *Il-6, TNF*α, and iNos was observed only at high concentrations, between 10 and 25 μM, but not at 1 μM in RAW264.7 macrophages (21). This suggests that the inhibition of Erk, a kinase known to be involved in the regulation of cytokine expression (46, 47), could mediate this effect. In addition, inhibition of Cdk7 by a specific inhibitor (BS-181) and siRNA-Cdk7 knockdown has already been demonstrated to reduce *IL-1*β*, IL-6, IL-8* transcript levels, and IL-1β/IL-6 secretion in LPS-induced MH7A cells (48), suggesting that the inhibition of Cdk7 may also be involved.

Since GCs are one of the most potent immunosuppressants and roscovitine was shown to be a potent anti-inflammatory drug, we further investigated whether a combination of roscovitine and Dex enhances the immunosuppressive effects on inflammatory mediator production in macrophages. Indeed, roscovitine in combination with Dex lead to stronger reduction of *Il-6, Il-1*β and *iNos* mRNA, and Il-6 and iNos protein expression after LPS induction. This finding suggests that a combinatorial treatment of roscovitine and Dex may be most beneficial for the treatment of inflammatory diseases. Furthermore, we demonstrate that the effect on inflammatory cytokines (*Il-6, Il-1*β) is additive and Cdk5 independent, suggesting that the immunosuppressive effect is mediated by independent pathways. Interestingly, for the iNos suppression roscovitine increased the anti-inflammatory potential of Dex after LPS induction. In addition, we showed that *Cdk5* deletion also led to a stronger reduction of iNos expression and NO production after LPS + Dex treatment. We still observed an albeit reduced roscovitine effect on *iNos* expression in *Cdk5* deficient LPS + Dex treated macrophages, suggesting an inhibition of additional kinases mediating the roscovitine effect. This suggests that roscovitine enhances the anti-inflammatory Dex effect on iNos mainly by Cdk5- independent mechanisms, which is in contrast to the general view where Cdk5 was shown to be a high-affinity target of roscovitine. Taken together, our results showed that the loss of Cdk5 potentiates the anti-inflammatory Dex effect on iNos and NO generation in inflammatory macrophages, a finding that has been not described so far.

### Specific *Cdk5* Deletion Has no Effect on Anti-inflammatory Markers in Macrophages

We also found, that *Cdk5* deletion had no major impact on the polarization of alternative (M2-like) macrophages as shown for example for *Il-10* expression after 24 h of stimulation. Seok et al. examined in detail the knockdown and knockout of *p35* (the *Cdk5* activator) and knockdown of *Cdk5* in LPS stimulated macrophages and showed that this enhances *Il-10* mRNA and Il-10 protein expression after 24 h (35). Moreover, we did not observe differences in the Dex-mediated induction of the M2 markers (*Cd163, Cd206, Ym1, Il-10*) upon Cdk5 deletion, except for *Mertk*, a phagocytosis marker, that was upregulated by Dex in the absence of *Cdk5*. Expression of other TAM receptors, such as *Tyro3, Axl*, and other phagocytosis receptors (*Cd36, Anxa1*) (49, 50) were not affected after *Cdk5* deletion in macrophages. Whether the deletion of *Cdk5* in macrophages increases the Dex-induced phagocytic capacity due to *Mertk* upregulation remains to be elucidated.

### *Cdk5* Regulates the Dex Effect on NO Production Through GR Phosphorylation, Mkp1, and p38Mapk During Inflammation

It is known that LPS increased iNos through p38Mapk in macrophages (44) and Dex reduced iNos expression and NO through destabilization of mRNA and increased iNos protein degradation by calpain (51–55). We therefore investigated the mechanism by which *Cdk5* deletion enhances the anti-inflammatory potential of Dex and suppresses iNos and NO production in pro-inflammatory macrophages.

The phosphorylation of GR at Ser211 was reduced in macrophages upon *Cdk5* deletion. *In vitro* kinase assays showed that Cdk5 phosphorylates the human GR at multiple serine residues (Ser203, Ser211, and Ser226) (41). In addition, Cdk5 phosphorylates GR at Ser211 and Ser203 in HCT116, Cos7, and rat cortical neuronal cells (41). This is in line with our observation showing reduced GR phosphorylation (Ser211) in macrophages lacking *Cdk5*. More recent *in vivo* studies suggested that Cdk5 is a crucial component of GR-dependent stress response in the brain by regulating GR phosphorylation (36, 56). To our knowledge, our data is showing for the first time that Cdk5-mediated GR phosphorylation (Ser211) is not restricted to the nervous system, but might also play an important role in innate immune cells like macrophages. Furthermore, the Cdk5-GR interaction seems to reduce GR activity especially for the target gene Mkp1.

Kino et al. reported an enhancement of mRNA expression for protein phosphatase 1 regulatory subunit 10 (*Ppp1r10*), the neuropeptide Y receptor (*Npy1r*), and serum and glucocorticoid-induced kinase (*Sgk*) in rat cortical neuronal cells, regardless of reduced GR phosphorylation upon Cdk5 inhibition (41). This is consistent with our results, which showed an induced Mkp1 expression but reduced GR Ser211 phosphorylation in the absence of *Cdk5* during Dex and LPS + Dex stimulation. Cdk5 was reported to contribute to an impaired GC-induced recruitment of the coactivators p300/CBP and SNF2 to the GRE-containing MMTV and endogenous Sgk promotors resulting in a reduced transcriptional activity (41). In addition, p300 was shown to act as an activator for Mkp1 expression (57). However, if coactivator recruitment is increased upon *Cdk5* deletion in macrophages remains to be addressed.

Mkp1 was shown to control Erk activation (58) and increased Mkp1 protein levels correlate with reduced Erk phosphorylation after 16 h of Dex treatment in mast cells (58). However, we did not observe genotype differences in Erk activation, but we did observe increased Mkp1 expression upon *Cdk5* deletion in macrophages after 4 h Dex treatment.

Mkp1 has also been reported to dephosphorylate p38Mapk and therefore contribute to the reduction of pro-inflammatory mediators (6, 58–63). In macrophage cell lines Mkp1 has already been described to negatively regulate iNos and NO production by inhibiting p38Mapk activity (45). Since we observed an upregulation of Mkp1 upon *Cdk5* deletion this could explain the lower levels of phosphorylated p38Mapk observed in LPS + Dex treated *Cdk5*^LysMCre^ macrophages. In line with this, *Cdk5*^LysMCre^ macrophages showed a stronger reduction in iNos and NO production during LPS + Dex treatment compared to *Cdk5*^flox^ macrophages. Furthermore, inhibition of p38Mapk confirmed a trend toward a potentiated repressive Dex effect on NO production after 48 h LPS + Dex treated Cdk5^LysMCre^ BMDMs. Thus, we conclude that upon *Cdk5* deletion the induction of Mkp1 leads to a stronger dephosphorylation of p38Mapk resulting in the reduced iNos and NO production in inflammatory macrophages. Our findings are further supported by the fact, that Mkp1^−/−^ mice are more sensitive to models of inflammatory diseases, such as sepsis and endotoxemia (6, 64), furthermore higher iNos expression was observed in the liver of Mkp1^−/−^ mice during sepsis (65). It should be mentioned, that roscovitine treatment did not reduce p38Mapk phosphorylation in wildtype macrophages during LPS treatment regardless of inflammatory cytokine inhibition. This is in line with previous work by Du et al. which showed enhanced p38Mapk phosphorylation upon roscovitine and LPS stimulation but reduced cytokine expression (21). Beyond this, p38Mapk was shown to phosphorylate GR at Ser211 (42, 51, 52, 66), therefore the reduced levels of phosphorylated p38Mapk upon Cdk5 deletion could be involved in the reduction of GR phosphorylation. Whether Cdk5 also influences metabolic GR target genes and therefore reduce or enhance severe side effects has not been investigated.

Here we report that roscovitine, a Cdk inhibitor, is a potent anti-inflammatory drug and combinatorial treatment with Dex leads to an additive suppression of pro-inflammatory mediator expression such as *IL-1*β and *Il-6*. However, we have shown, that roscovitine synergistically with Dex suppresses iNos induction in inflammatory macrophages. Furthermore, we have demonstrated by generating Cdk5 conditional knockout mice that *Cdk5* deletion is sufficient to enhance the anti-inflammatory effect of Dex on iNos. Since roscovitine also exerts its immunosupressive effect in *Cdk5* deficient macrophages albeit to a lesser degree, the effects of roscovitine inhibition is mainly mediated by the inhibition of other Cdks than Cdk5. Macrophage specific *Cdk5* deletion reduced Dex-dependent GR Ser211 phosphorylation, but induced Mkp1 expression and reduced p38Mapk phosphorylation hence resulting in a decrease of iNos and NO production. Here we have shown a novel mechanism of Cdk5 involved in the anti-inflammatory effects of GCs. In summary, this study supports the use of combinatorial treatment of inflammatory diseases with specific Cdk5 inhibitor(s) and GCs to potentiate the anti-inflammatory effect on iNos and NO. Furthermore, combinatorial treatment may be a possible therapeutic objective to lower GC doses and therefore avoid negative side effects.

## Data Availability

All datasets generated for this study are included in the manuscript and/or the Supplementary Files.

## Ethics Statement

This study was carried out in accordance with the recommendations of Tierschutzgesetz and Tierschutz-Versuchstierordnung (Mitteilung nach § 4), Regierungspräsidium Tübingen, Baden-Wurrtemberg. The protocol was approved by the Regierungspräsidium Tübingen, Baden-Wurrtemberg.

## Author Contributions

PP, MF, UB, LL, and SV performed experiments. MF revised the manuscript. PP and SV designed the study, performed experiments, processed the data, and wrote the manuscript.

### Conflict of Interest Statement

The authors declare that the research was conducted in the absence of any commercial or financial relationships that could be construed as a potential conflict of interest.
