# Supplementary material for: Cdk5 Deletion Enhances the Anti-inflammatory Potential of GC-Mediated GR Activation During Inflammation
Source: Front Immunol. 2019 Jul 10;10:1554. doi: 10.3389/fimmu.2019.01554 (PMC6635475; doi:10.3389/fimmu.2019.01554)
Supplement: Supplementary file 1 [file Table_1.DOCX]

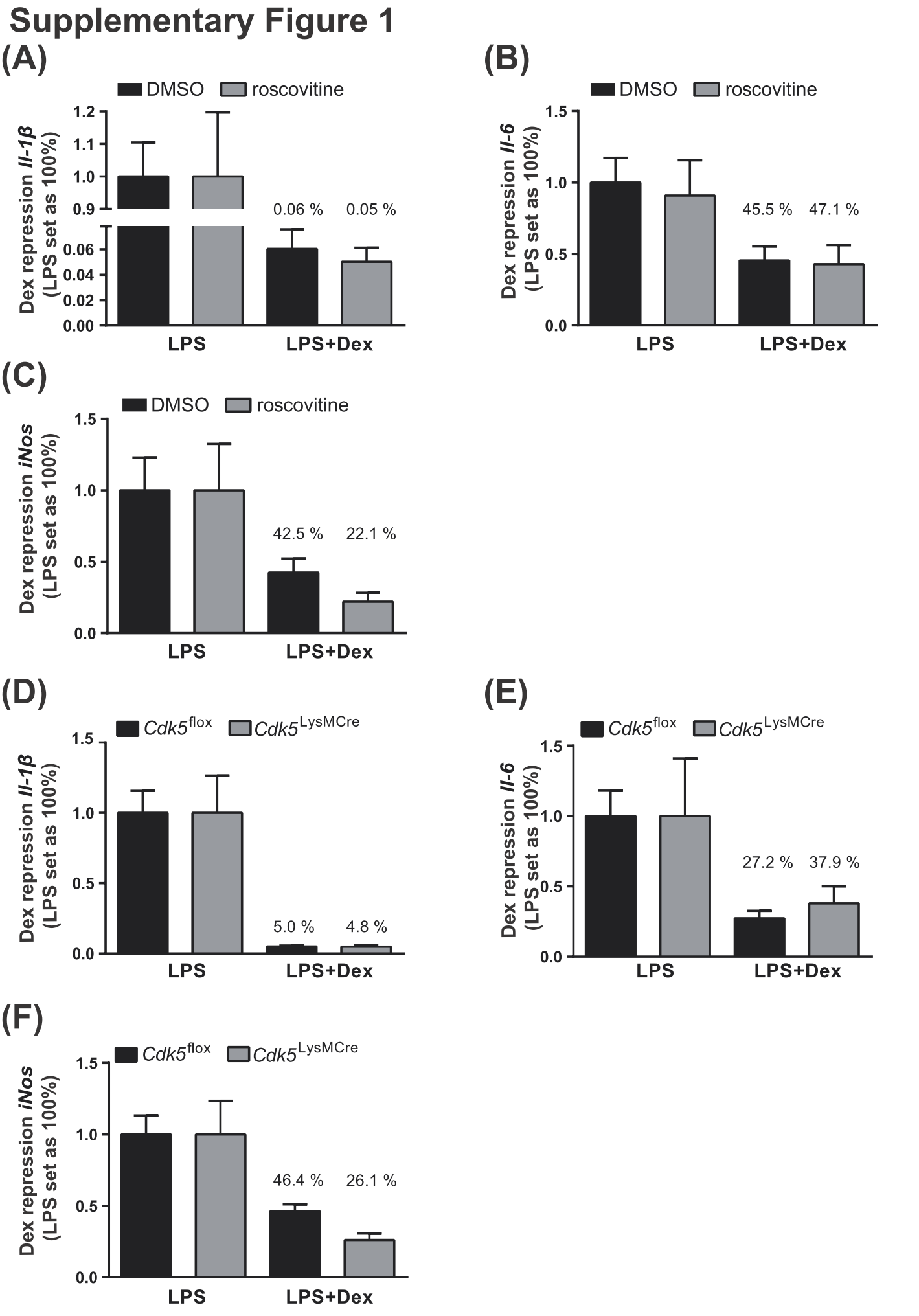


**Supplementary Figure 1**

**(A-C)** 4h LPS treatment of BMDMs derived from wildtype mice in the presence of either DMSO or 10 µM roscovitine were both set as 100% to examine the suppressive Dex effect on **(A)** *Il-1β* mRNA expression, **(B)** *Il-6* mRNA expression and **(C)** *iNos* mRNA expression after 4h combinatorial treatment. Data are taken from Figure 1A-C. **(D-F)** 4h LPS treatment of BMDMs derived from *Cdk5*^flox^ and *Cdk5*^LysMCre^ were both set as 100% to examine the suppressive Dex effect on **(D)** *Il-1β* mRNA expression, **(E)** *Il-6* mRNA expression and **(F)** *iNos* mRNA expression after 4h combinatorial treatment. Data are taken from Figure 2A-C.

**Supplementary Figure 2**


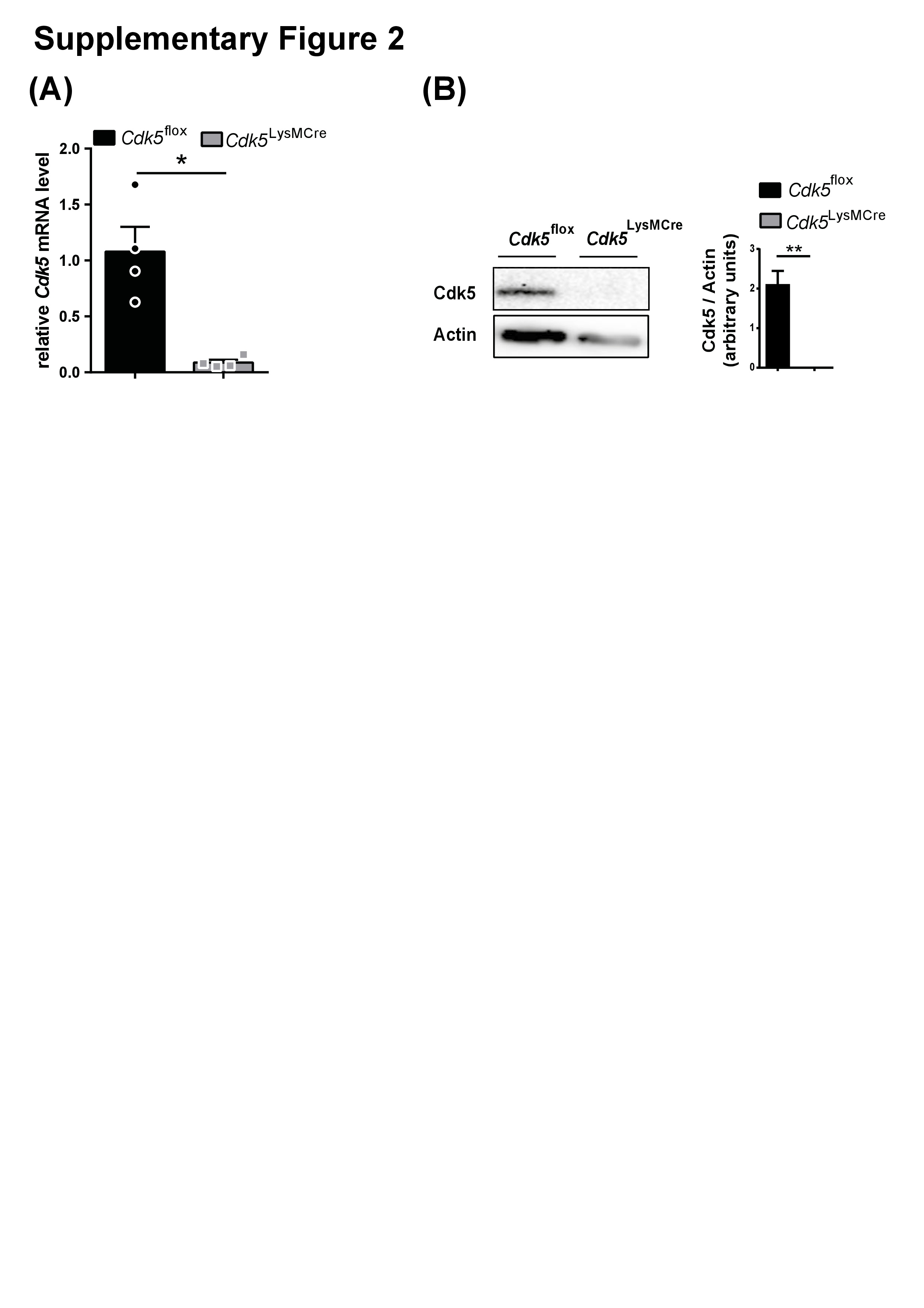


**(A)** The relative mRNA expression of *Cdk5* in BMDMs derived from *Cdk5*^flox^ and *Cdk5*^LysMCre^ mice was determined by qRT-PCR in 4h PBS-treated BMDMs. **(B)** Cdk5 protein (30 kDa) was detected by western blot in 4h PBS-treated BMDMs from *Cdk5*^flox^ and *Cdk5*^LysMCre^ mice and quantified. β-Actin (43 kDa) served as loading control. Data shown in A: n = 4 and B: n = 3. Results are depicted as mean ± SEM. Statistical analysis was performed by Wilcoxon-Mann-Whitney test (two-tailed) * p ˂ 0.05; ** p ˂ 0.01; n.s. not significant.


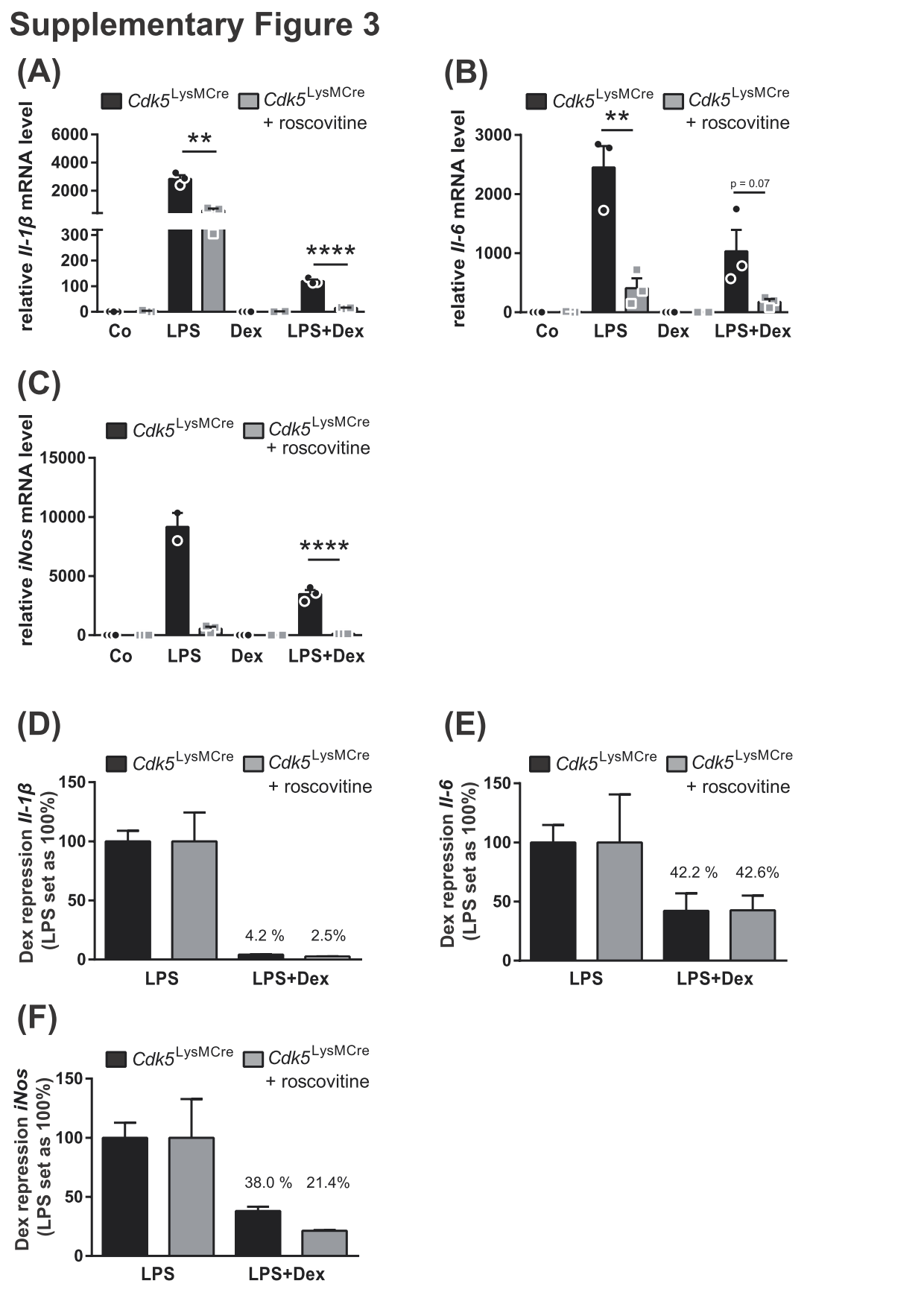


**Supplementary Figure 3**

**(A-C)** BMDMs derived from *Cdk5*^LysMCre^ mice were stimulated 4h with PBS (Co), LPS (100 ng / ml), Dex (10^-6^ M) or a combination of LPS + Dex with 30 min pre-treatment of either DMSO or 10 µM roscovitine. **(A)** Relative *Il-1β* mRNA expression, **(B)** relative *Il-6* mRNA expression, **(C)** relative *iNos* mRNA expression were analyzed by qRT-PCR after 4h. **(D-F)** 4h LPS treatment of BMDMs derived from *Cdk5*^LysMCre^ treated with DMSO or 10 µM Roscovitine were both set as 100% to examine the suppressive Dex effect on **(D)** *Il-1β* mRNA expression, **(E)** *Il-6* mRNA expression and **(F)** *iNos* mRNA expression after 4h combinatorial treatment. Data are taken from Supplementary Figure 3A-C. Data shown in A: n = 3, B: n = 3, C: n = 3 (instead of *Cdk5*^LysMCre^ LPS treatment n = 2, therefore no statistical analysis could be applied). Results are depicted as mean ± SEM. Statistical analysis was performed by t-test (two-tailed). ** p ˂ 0.01; **** p ˂ 0.0001.


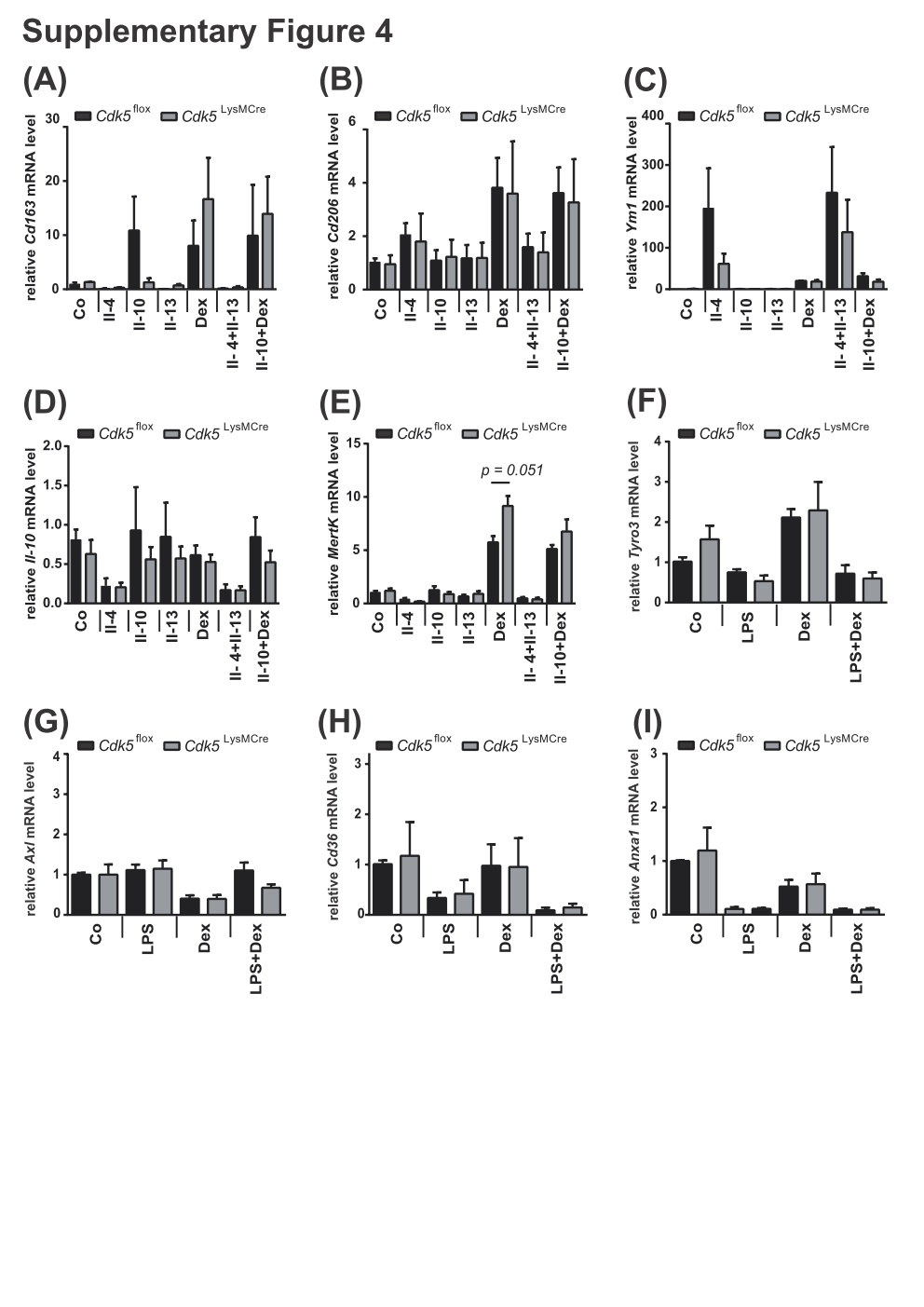


**Supplementary Figure 4**

**(A-E)** BMDMs from *Cdk5*^flox^ and *Cdk5*^LysMCre^ mice were induced to alternative activated macrophages (M2-like) with Il-4 (20 ng / ml), Il-10 (20 ng / ml), Il-13 (20 ng / ml), Dex (10^-7^ M), Il-4 (20 ng / ml) + Il-13 (20 ng / ml) and Il-10 (20 ng / ml) + Dex (10^-7^ M) for 24h. **(A)** Relative *Cd163* mRNA expression, **(B)** relative *Cd206* mRNA expression, **(C)** relative *Ym1* mRNA expression, **(D)** relative *Il-10* mRNA expression and **(E)** relative *Mertk* mRNA expression were measured after 24h. **(F-I)** BMDMs from *Cdk5*^flox^ and *Cdk5*^LysMCre^ mice were treated with PBS (Co), LPS (100 ng / ml), Dex (10^-7^ M) or LPS (100 ng / ml) + Dex (10^-7^ M) and **(F)** relative *Tyro3* mRNA expression, **(G)** relative *Axl* mRNA expression, **(H)** relative *Cd36* mRNA expression and **(I)** relative *Anxa1* mRNA expression were measured with qRT- PCR after 24h treatment. Data shown in A-I: n = 3-4. Results are depicted as mean ± SEM. Statistical analysis was performed by Wilcoxon-Mann-Whitney test (two-tailed).


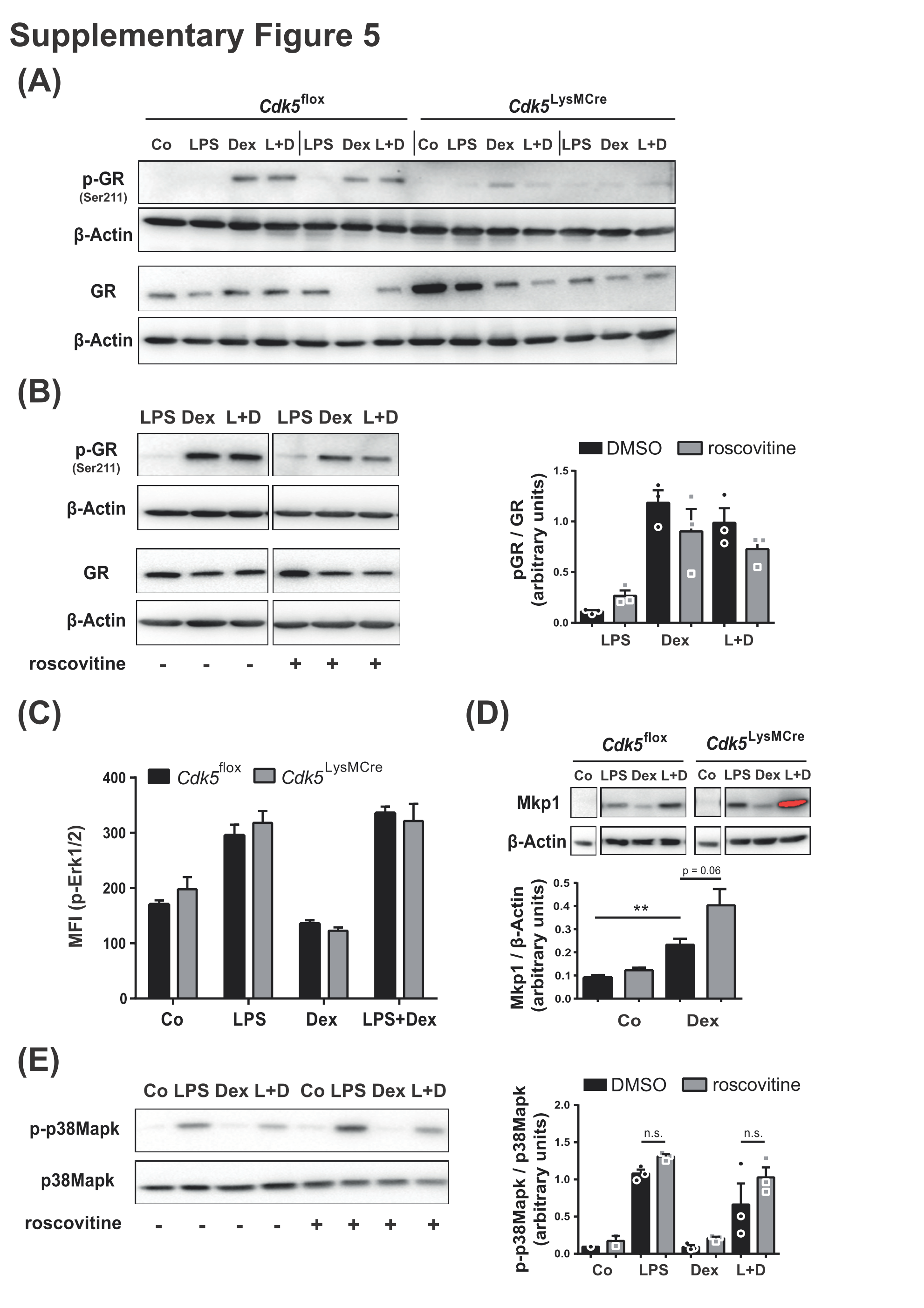


**Supplementary Figure 4**

**(A)** BMDMs derived from *Cdk5*^flox^ and *Cdk5*^LysMCre^ mice were stimulated with PBS (Co), LPS (100 ng / ml), Dex (10^-6^ M) or a combination of LPS + Dex (L+D) for 4h and phosphorylated GR (Ser211) protein (95 kDa) and total GR protein (94 kDa) was detected by western blot on two separate gels. β-Actin (43 kDa) served as loading control on the individual gels. **(B)** BMDMs derived from wildtype mice were stimulated 4h with LPS (100 ng / ml), Dex (10^-6^ M) or a combination of LPS + Dex with 30 min pre-treatment of either DMSO or 10 µM roscovitine and phosphorylated GR (Ser211) protein (95 kDa) and total GR protein (94 kDa) was detected by western blot on two separate gels. β-Actin (43 kDa) served as loading control on the individual gels. Quantification of 3 blots is shown. **(C)** phospho-Erk1/2 (Thr202/Tyr204) was detected by median fluorescence intensity (MFI) with the Bio-Plex Pro™ cell signaling MAPK-Panel. **(D)** BMDMs were treated as described in (A) Mkp1 protein (40 kDa) was detected by western blot after 4h. β-Actin (43 kDa) served as loading control. Blot (from Figure 3D) was overexposed to show the Dex effect on Mkp1. Only control and Dex lanes were used for quantification. All samples were at the same blot. **(E)** BMDMs derived from wildtype mice were stimulated 4h with PBS (Co), LPS (100 ng / ml), Dex (10^-6^ M) or a combination of LPS + Dex with 30 min pre-treatment of either DMSO or 10 µM Roscovitine and phospho-p38Mapk (43 kDa) and p38Mapk (40 kDa) protein were detected by western blot after 4h and quantified. Data shown in B: n = 3; C: n = 7; D: n = 5; E: n = 3. Results are depicted as mean ± SEM. Statistical analysis was performed by Wilcoxon-Mann-Whitney test (two-tailed). ** p ˂ 0.01, n.s. not significant.
